# Increasing use of mental health services in remote areas using mobile technology: a pre–post evaluation of the SMART Mental Health project in rural India

**DOI:** 10.7189/jogh.07.010408

**Published:** 2017-06

**Authors:** Pallab K Maulik, Sudha Kallakuri, Siddhardha Devarapalli, Vamsi Krishna Vadlamani, Vivekanand Jha, Anushka Patel

**Affiliations:** 1 The George Institute for Global Health, New Delhi, India; 2 The George Institute for Global Health, University of Oxford, Oxford, United Kingdom; 3 The George Institute for Global Health, University of Sydney, Sydney, Australia

## Abstract

**Background:**

About 25% of the Indian population experience common mental disorders (CMD) but only 15–25% of them receive any mental health care. Stigma, lack of adequate mental health professionals and mental health services account for this treatment gap, which is worse in rural areas. Our project evaluated task shifting and mobile–technology based electronic decision support systems to enhance the ability of primary care health workers to provide evidence–based mental health care for stress, depression, and suicidal risk in 30 remote villages in the state of Andhra Pradesh, India.

**Methods:**

The Systematic Medical Appraisal Referral and Treatment (SMART) Mental Health project between May 2014 and April 2016 trained lay village health workers (Accredited Social Health Activists – ASHAs) and primary care doctors to screen, diagnose and manage individuals with common mental disorders using an electronic decision support system. An anti–stigma campaign using multi–media approaches was conducted across the villages at the outset of the project. A pre–post evaluation using mixed methods assessed the change in mental health service utilization by screen positive individuals. This paper reports on the quantitative aspects of that evaluation.

**Results:**

Training was imparted to 21 ASHAs and 2 primary care doctors. 5007 of 5167 eligible individuals were screened, and 238 were identified as being positive for common mental disorders and referred to the primary care doctors for further management. Out of them, 2 (0.8%) had previously utilized mental health services. During the intervention period, 30 (12.6%) visited the primary care doctor for further diagnosis and treatment, as advised. There was a significant reduction in the depression and anxiety scores between start and end of the intervention among those who had screened positive at the beginning. Stigma and mental health awareness in the broader community improved during the project.

**Conclusions:**

The intervention led to individuals being screened for common mental disorders by village health workers and increase in mental health service use by those referred to the primary care doctor. The model was deemed feasible and acceptable. The effectiveness of the intervention needs to be demonstrated using more robust randomized controlled trials, while addressing the issues identified that will facilitate scale up.

Mental disorders is a major global public health problem and accounts for 8.5% of the total years of life lost due to premature death and years lived with disability globally [1]. Between 13–50% of Indians suffer from common mental disorders (CMD) such as depression, stress and suicidal risk [2], but majority receive little or no care, with estimates from low– and middle–income countries (LMICs), such as India, suggesting that only 15–25% of affected individuals receive any treatment for their mental illness [3], resulting in a large ‘treatment gap’. The reasons for this gap are numerous, but include poor awareness about mental health, personal and community stigma related to mental illness and help seeking, lack of appropriate mental health services and trained mental health professionals [4,5]. Treatment gaps are more in rural populations [6], especially in Scheduled Tribe (ST) communities in India, which have particularly poor infrastructure and resources for health care delivery in general, and almost no capacity for providing mental health care.

The ST communities are identified as culturally or ethnographically unique by the Indian Constitution. They are populations with poorer health indicators and fewer health care facilities compared to non–ST rural populations, even when within the same state [7], and often live in demarcated geographical areas known as ST areas. In Andhra Pradesh, the state where the current study is based, infant mortality rate in ST areas and non ST areas was 94.1 and 54.0, respectively; and under–5 mortality rate was 112 and 63.2, respectively [7]. Primary health care systems in ST areas are similar to those available in other rural areas with a tiered model involving sub–centers, primary health centers (PHCs) and district hospitals. Non–physician health care workers, called Accredited Social Health Activists (ASHAs), are a key resource for providing health care in rural settings. These female health care workers are community members with an average of 8–10 years of formal education, who mainly focus on maternal and child health by visiting households regularly and systematically. Each ASHA is responsible for about 1000 villagers. Primary care doctors are in–charge of the activities of a PHC which covers 20 000–30 000 population. They provide primary health care and refer any condition that they cannot manage, including mental disorders, to the next level of care at district hospitals.

One way to reduce treatment gap is by addressing the lack of trained mental health professionals in rural areas by using task shifting and training primary care health workers in the villages to manage CMD. A systematic review found that task shifting involving non–physician health workers is beneficial for a number of chronic conditions including mental disorders [8]. The process can be facilitated by using screening and management protocols that could be used by them easily. Task shifting for ASHAs and primary care doctors involve training and provision of basic skills to identify and manage CMD, which otherwise would have been the responsibility of trained mental health professionals. Task shifting for ASHAs involves training on concepts about mental health and screening for CMD; and providing skills in basic mental health care. Task shifting for primary care doctors involve training in interviewing, diagnosing, and managing CMD using standardized guidelines and algorithms.

Integrating standardized protocols into algorithm based electronic decision support systems (EDSS) could facilitate task shifting, by making the protocols easier to administer. A number of systematic reviews have outlined the effectiveness of EDSS to deliver appropriate health care [9–13]. Computer–based decision support systems are effective in bringing about positive change [14], and provision of individualised recommendations using an EDSS have been found to be useful [12]. Mobile technology based EDSS using commercial mobile networks leverages the increasing penetration of mobile phones across India, including rural India, increasing 3G connectivity that allows faster data sharing, and availability of cheaper smart phones/tablets. Prior research has highlighted the use of mHealth in communicable diseases and maternal and child health, but its research on use in mental illness is limited [15].

Another way to reduce treatment gap and increase demand for mental health services is by increasing knowledge about mental health and reducing stigma related to mental illness and help–seeking [4,5,16]. Research has shown that interventions especially those involving social contact with people with mental illness are effective in reducing stigma [17,18].

This paper reports on a “proof of concept” project – Systematic Medical Appraisal Referral and Treatment (SMART) Mental Health project – conducted in rural Andhra Pradesh, India. The project used task shifting supported by a mobile technology based mental health services delivery model for screening, diagnosing, and managing CMD. A campaign to increase mental health awareness and reduce stigma related to mental health and help–seeking was also implemented as part of the intervention. The aim was to ascertain the acceptability, feasibility and preliminary impact of the intervention, specifically on mental health services use, using mixed evaluation methods. This paper focuses on the key quantitative results.

## METHODS

The methods used have been outlined earlier [19], and are summarized below.

### Objectives

The project had two key objectives:

1. The development of a multifaceted intervention using training, task shifting, and mobile–based decision support to increase the screening and referral of individuals with CMD in one area of rural Andhra Pradesh.
2. To evaluate the feasibility and acceptability of the intervention amongst community members, health workers, and other stakeholders, and document preliminary evidence and lessons learned about the intervention for future study and scale up.

The primary outcome of the evaluation was to assess the change in proportion of mental health services use by individuals who were suffering from CMD. Other quantitative outcomes included changes in depression and anxiety scores, number of individuals screened, and number of screen positive individuals referred to the primary care doctor. The feasibility and acceptability of the intervention including process evaluation were assessed using qualitative interviews conducted at post–intervention, and will be reported separately.

### Site

The project was implemented in 30 villages associated with two PHCs, located in an ST area of the West Godavari district of Andhra Pradesh. All villages served by the PHCs were listed and a random selection of 15 villages from each PHC was made. The eligibility criteria for selecting villages were that all villages should have ASHAs and the population should be proportionate to the number of ASHAs. One village with a population >3000, was replaced by another one with a smaller population, keeping in mind that the average population was ~ 400. The main source of livelihood was farming. In Andhra Pradesh, 5.5% of the total population belong to ST communities. The West Godavari district of Andhra Pradesh, where this project was conducted, has 3.4% ST population living in ST areas (http://aptribes.gov.in/statistics.htm), which are in the interior or more remote areas of the district. On extrapolating recent Census data from 2011 (http://censusindia.gov.in/pca/cdb_pca_census/Houselisting–housing–AP.html) and data collected through the government led Anganwadi program, more than two–third of the population belonged to the ST communities and other backward communities in the 30 villages. All eligible adults ≥18 years of age who gave consent to participate, were able to understand the questions and instructions, and were not limited by any severe physical disorder from accessing mental health services were invited to participate.

### Duration

24 months, from May 2014 to April 2016.

### Ethical considerations

Ethics approval was received from the Independent Review Committee of the Centre for Chronic Disease Control, New Delhi, India. Informed written consent was obtained from all participants and data were collected according to the Declaration of Helsinki on ethical research. Approval for conducting the project was obtained from the Health Department, Government of Andhra Pradesh, and the Integrated Tribal Development Agency was informed about the project. Approval was also obtained from all the local village administrations. Data are reported as per STROBE (Strengthening the Reporting of Observational Studies in Epidemiology) guidelines for reporting observational studies [20].

### Development of the multifaceted intervention

#### 1. Development of mobile technology based EDSS

A single screening and management algorithm was developed for use by the doctors and ASHAs. The screening tool used by ASHAs was based on standard screening tools – Patient Health Questionnaire (PHQ9) [21] and Generalized Anxiety Disorder questionnaire (GAD7) [22]. The diagnosis and management guidelines used by the doctors was based on the Mental Health Gap – Intervention Guide (mhGAP–IG) [23]. The system was developed on an OpenMRS platform and allowed clinical data to be shared between the ASHA and doctor using cloud computing. The algorithm and user interface were programmed for use as an application on a 7 inch Android tablet. Both the PHQ9 and GAD7 provide diagnoses of mild/ moderate/ severe levels of depression/anxiety based on scores 5–9, 10–14. ≥15, respectively [24]. Only scores ≥10 on either scale, or a positive response to the question on self–harm in the PHQ9, were considered as screen positive for this project. As anxiety is commonly associated with depression, GAD7 scores were also considered as indicative of depression. The tool for the ASHAs was developed in Telugu, whereas the mhGAP–IG tool was in English. The algorithms were developed for a mobile platform and finalized iteratively using simulated data. Subsequently, mock clinical data were validated against a psychiatrist’s diagnosis. As a final step, the applications were tested in one village as part of a formative process, for user acceptability and identification of issues related to functionality [25].

#### 2. Interactive voice response system (IVRS)

An algorithm based IVRS sent out pre–recorded messages to the screen positive individuals to continue care as advised by the ASHA or the doctor; and to the ASHAs and doctors to screen and followup individuals as per guidelines.

#### 3. Stigma Reduction Campaign

A stigma reduction campaign was conducted at the outset to increase knowledge about mental health in the community and reduce stigma related to mental health. This was perceived as a key step to ensure that the services were availed and the importance of CMD was understood. The campaign’s objective was to increase mental health knowledge and reduce stigma in the community, and was conducted for 8 weeks prior to the baseline survey across all villages. It included a number of strategies: sharing brochures and posters on mental health awareness and information with the community using a door–to–door campaign; showing a video of a person talking about his own mental illness and a video of a film actor talking about CMD; staging live performances or video recordings of a drama on mental disorder and help–seeking. Two instruments were used; the first was the Knowledge, Attitude and Behaviour about mental health instrument [26] and the second was Barriers to Access to Care Evaluation – Treatment Stigma Subscale (BACE–TS) [27]. They were administered at baseline and at post–intervention. They are a 16–item and 12–item questionnaire, respectively, with Likert type responses. Mean scores for each item can be calculated, with lower scores indicating lower knowledge and lower stigma, respectively.

### Demonstrating feasibility, acceptability and potential impact of the intervention

#### 4. Baseline household survey (September – October 2015)

A baseline survey of the whole community (all households in all 30 villages) was conducted by trained interviewers using tablets. The survey enquired about socio–demographic details, stressors, social network, CMD, past history of mental disorders and its treatment, family history of mental disorders, and perceptions about stigma related to mental health.

#### 5. Intervention (3 months– November 2015–January 2016)

Both ASHAs and PHC doctors were provided training on identification and management of CMD using EDSS. The ASHAs received classroom training from research staff for one week and then received additional supervised field training for an additional day, followed by feedback. The total period of training was 10 days. Concepts conveyed included mental health, CMD, treatment needs and a basic understanding about treatment modalities using medicines or counselling. Each doctor was trained individually by the Principal Investigator for one day and then additional support was provided to them by the field staff to sensitize them to the tablets and application. The doctors were trained using the mhGAP modules and were provided guidance on interviewing skills, diagnosis, and treatment guidelines. For both ASHAs and doctors, the research staff provided continued support and feedback on the tools over the whole course of the study on a needs basis. The ASHAs used the PHQ9/GAD7 to screen the population in their homes for CMD. They referred all those who screened positive to the PHC doctor. The doctor used another EDSS based on the mhGAP–IG tool to diagnose and manage those cases, either at PHCs or at health camps organized in villages. The mhGAP–IG tool provided the doctors a suite of recommended treatments for managing patients they diagnosed with CMD. The doctors followed those recommendations to provide treatment as needed. Health camps were organized in selected villages, so that patients could visit the doctor closer to home. The PHC doctor and ASHAs were available at this camp. The doctor not only saw patients with CMD, but also any other medical conditions. People from all neighbouring villages were asked to visit the health camp and ASHAs informed people screened positively for CMD about the time and location of the camp. Individuals with severe mental disorders or any other complications were referred to mental health professionals at district hospitals. Treatment data were shared electronically between the ASHAs and the PHC doctors, and an algorithm–based mechanism helped ASHAs to plan follow–up schedules and prioritize individuals who needed prompt follow–up to ensure treatment adherence. The screen positive individuals, ASHAs and doctors also received IVRS messages to facilitate followup and treatment adherence. The project staff monitored the work of ASHAs and doctors and responded to any project or application specific problems faced by them.

#### 6. Post–intervention phase (February – March 2016)

All screen–positive individuals identified by ASHAs were re–interviewed at the end of the intervention by trained interviewers, using a questionnaire. Process evaluation of the project was done using focus group discussions and in–depth interviews of key stakeholders – community members, ASHAs, primary care doctors, village leaders and field staff – to identify barriers and facilitators in implementing the project.

### Data management and statistical analyses

All data were captured electronically, encrypted and stored on secure servers at the George Institute office in Hyderabad. All tablets and servers were password protected. Data on tablets could be accessed by user defined login. Additionally all applications were locked by the administrator so that only data and applications relevant to the project were accessible to ASHAs and doctors, and once data were submitted by ASHAs or doctors it could be changed only by the administrator in case of any errors. All data were cleaned by the data management team. Most of the coding was predefined at the time of developing the electronic data capture tools by the software developers in consultation with the researchers. Further modifications, as per need, were made by researchers. Only de–identified data were shared with researchers for analyses.

### Sample size calculation

Villages in the ST areas are smaller in size compared to other rural villages in the West Godavari district; for 30 villages we estimated the population would be around 10 000. We anticipated that approximately 7500 individuals will be aged ≥18 years based on the demographic profile. Based on our extensive previous work, we expected a response rate of 75%, or about 5600 participants. It was conservatively estimated that about 15% of consenting participants at baseline will have a CMD as determined by the screening tools, representing approximately 850 individuals. Studies have estimated that in developing countries only 15–25% of those with severe mental disorders receive treatment, and these numbers are even less for CMD [4]. We conservatively assumed that 10% of individuals who screened positive will have sought medical care for mental disorders in the previous 12 months at baseline.

With these assumptions, a project involving 360 screen positive individuals would have 80% power at 2–tailed α = 0.05, to detect a relative increase of mental health care utilization by as little as 30% (ie, from 10% to 13%) at follow–up. This further assumes up to 4% of discordant results between baseline and follow–up; that is, up to 0.5% who switch from utilizing services at baseline to no longer accessing services at follow–up. Other studies on provision of mental health services in primary care in India have found an intracluster correlation (ICC) of 0.03 [28]. After adjusting for the ICC and 30 clusters/villages a sample size of 545 individuals (on average 18 per cluster) was estimated to provide 80% power.

### Outcomes of interest

The proportionate change in mental health services use following intervention was the primary outcome. Depression and anxiety scores among those who had scored ≥10 (cut off score for screening positive) on either the PHQ9 or GAD7 at the beginning of the intervention were compared with their scores at post–intervention phases using paired t–test. The cluster impact (village as a cluster) was explored by adding village as a random effect to a mixed model of change from baseline anxiety and depression score.

## RESULTS

**Table 1** outlines the population base and participants screened at different phases of the project. Training was provided to 21 ASHAs and 2 PHC doctors in managing CMD.

**Table 1:** Population screened at different stages of the project

Stage of project	Population screened at each stage	n/N (%)
Total population in the villages	10676	–
Total adult population (≥18 year)	8182	8182/10676 (76.6)
Baseline survey:		
Adult population contacted during baseline survey	7152	7152/8182 (87.4)*
Adult population screened at baseline following informed consent	5167	5167/7152 (72.2)†
Population who “screened positive” at baseline	339	339/5167 (6.6)
Screening at start of intervention:		
Adult population screened by ASHAs during intervention	5007	5007/5167 (96.9)
Adult population who ‘screened positive’ at intervention	238	238/5007 (4.8)

ASHAs – Accredited Social Health Activists

*Those not contacted had moved out of the village in search of seasonal jobs and none in the household could be identified.

†Reasons for not being screened were that even after 3 attempts they could not be contacted because they had temporarily moved out of the village and were unavailable; had died or were unwell; people less than 18 y were misclassified during the listing process as adults; 8 people refused.

The socio–demographic and basic health parameters of the 5167 individuals screened at baseline is presented in **Table 2**. There were 775 (15.0%) individuals with a score of ≥5 for either depression/anxiety, corresponding to mild to severe depression/anxiety. At baseline, 200 (3.9%) individuals had a score of ≥10 on the depression/anxiety scale corresponding to moderate/severe types of depression/anxiety, and 224 had responded positively to the question on suicide. All individuals who screened positive were advised to seek medical care from the nearest doctor or health facility (as the intervention had not been deployed at this time). Family members of those who screened positive for suicide were specifically told about the risk and the need for seeking urgent medical care, after obtaining permission from the individual. Eighty–five (42.5%) individuals out of the 200 who had a score of ≥10 on the depression/anxiety scale, had responded positively to the question on suicide.

**Table 2:** Socio–demographic and health characteristics of baseline population (N = 5167)

Characteristic	Baseline – n (%)
Gender:	
**Gender:**	
Female	3026 (58.56)
Male	2141 (41.44)
**Occupation:**	
Unorganized sector*	3706 (71.72)
Organized sector	224 (4.34)
Housewife/retired	887 (17.17)
Other	350 (6.77)
**Education:**	
No school	2408 (46.60)
Primary school	1438 (27.83)
High school	938 (18.15)
Graduate/post–graduate	362 (7.01)
Other	21 (0.41)
**Marital status:**	
Never married	741 (14.34)
Currently married	3882 (75.13)
Separated/divorced/widowed	544 (10.53)
**Age (years):**	
Mean (SD)	39.7 (14.69)
Range	18–92
**Past history of physical/mental illness based on doctor’s diagnosis:**	
Angina	139 (2.69)
Stroke	75 (1.45)
Diabetes	232 (4.49)
Cancer	7 (0.14)
Mental disorder	27 (0.52)
Family history of mental illness:	
Presence of a family history	95 (1.84)
**Substance use in lifetime:**	
Tobacco (cigarettes, bidi, gutka, cigars, etc.)	1502 (29.07)
Alcohol (beer, wine, spirits, etc.)	1781 (34.47)
Others (cannabis, cocaine, opioid, sedatives, hallucinogens, amphetamine, inhalants)	30 (0.59)
**Severity of depression (PHQ9):**	
Score 5–9 (mild)	442 (8.55)
Score 10–14 (moderate)	98 (1.90)
Score ≥15 (severe)	55 (1.06)
Severity of anxiety (GAD7):	
Score 5–9 (mild)	367 (7.10)
Score 10–14 (moderate)	82 (1.59)
Score ≥15 (severe)	22 (0.43)
**Stressful events (number):**	
0	2891 (55.95)
1	1527 (29.55)
2–3	692 (13.39)
≥4	57 (1.10)

*Agricultural laborer, manual laborer, skilled worker, farmer and business are reported under unorganized sector.

The commonest stressful events in the past year, associated with moderate or severe depression/anxiety, were suffering financial problems, suffering from major illness/injury either to self or loved ones, and death of loved ones (**Table 3**).

**Table 3:** Summary of stressful events faced by individuals who had a depression or anxiety score ≥10 at baseline (N = 200)

Question	Baseline n (%)
Did you get married in the last 1 year?	2 (1.0)
Did you get separated/divorced in the last 1 year?	1 (0.5)
Did your spouse die in the last 1 year?	6 (3.0)
Did any of your loved ones die in the last 1 year?	43 (21.5)
Did you have a baby in the last 1 year?	2 (1.0)
Did you lose your job in the last 1 year?	2 (1.0)
Did you retire in the last 1 year?	2 (1.0)
Did you or your loved one suffer any major illness/injury in the last 1 year?	48 (24.0)
Did you have any problems with your boyfriend/girlfriend in the last 1 year?	21 (10.5)
Did you have any major problems with your school/college performance in the last 1 year?	4 (2.0)
Did you have any major financial problems in the last year?	97 (48.5)
Did you face any natural disaster or stolen livestock or death of livestock, or crop failure or forced migration leading to loss of income or property?	36 (18.0)
Did you experience any major crime or were a victim of a major crime like robbery, assault/beating, murder/attempted murder, sexual violence?	15 (7.5)

During the intervention, the ASHAs re–screened all eligible adults and identified 238 individuals (4.75%) as ‘screen positive’ (72.7% female). There were only 38 individuals who were commonly identified as screen positive at both the baseline survey by interviewers and screening done by ASHAs at the beginning of the intervention.

**Figure 1:**
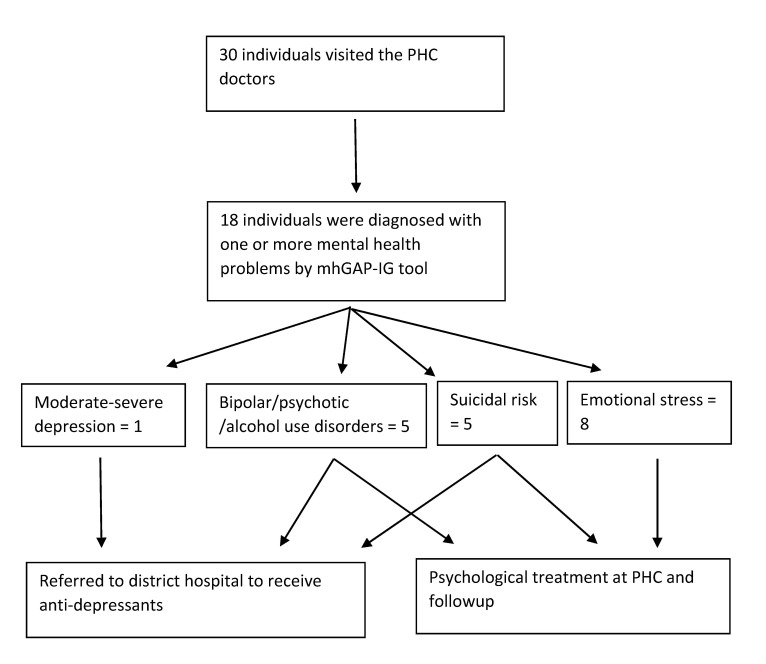
Final diagnosis of screen positive individuals and treatment provided.

Thirty of the individuals identified as screen positive by ASHAs visited a doctor, with 19 visiting camps and 11 visiting the PHCs. Of these, 24 (80%) were female. Eighteen of these 30 individuals were diagnosed with a confirmed mental health problem by the mhGAP–IG tool (**Figure 1**). Due to non–availability of anti–depressant medication, the individual with moderate depression was referred to the district hospital. Psychological therapy consisting of discussions on stressors, involving social networks, participating in pleasurable activities and work were provided to all individuals with emotional stress, 2 individuals with suicidal risk, and one with bipolar disorder. They were all asked to follow-up later to assess progress. Others were referred to the district hospital for specialist care. The ASHAs completed a follow–up visit for almost 80% of screen positive individuals.

Only 2 (0.8%) out of the 238 screen positive individuals had received mental health treatment in the past. The increase in mental health service use in this population was from 0.8% at the beginning of intervention to 12.6% at the end of intervention. Due to the small numbers, we had not adjusted for the clustering effect of villages for this analysis.

Post–intervention data were collected from 232 out of the 238 individuals who had screened positive during the intervention (**Table 4**). Three people had died due to natural causes (none due to self–harm) and 3 could not be contacted even after repeated attempts.

**Table 4:** Characteristics of the screen–positive population identified by ASHAs (N = 238)*

Characteristic	Post intervention – n/N (%)
**Gender:**	
Female	169/238 (71.01)
Male	63/238 (26.47)
Missing	6/238 (2.52)
**Occupation:**	
Unorganized sector	169/238 (71.01)
Organized sector†	9/238 (3.78)
Housewife/retired	41/238 (17.23)
Other	13/238 (5.46)
Missing	6/238 (2.52)
**Education:**	
No school	144/238 (60.50)
Primary school	52/238 (21.85)
High school	21/238 (8.82)
Graduate/Post–graduate	13/238 (5.46)
Other	2/238 (0.84)
Missing	6/238 (2.52)
**Marital status:**	
Never married	20/238 (8.40)
Currently married	173/238 (72.69)
Separated/divorced/widowed	39/238 (16.39)
Missing	6/238 (2.52)
**Age (years):**	
Mean (SD)	44.1(14.83)
Range	19 – 92

ASHAs – Accredited Social Health Activists

*Out of 238 at the beginning of the intervention stage, 232 were interviewed at the post–intervention stage; out of the 6 missing at post–intervention stage 4 were women, all 6 had either no schooling or only primary levels schooling, 5 worked in the unorganized sector and 1 was housewife/retired, all were married, and the average ages were similar to that of the larger group

†Agricultural laborer, manual laborer, skilled worker, farmer and business are reported under unorganized sector.

**Table 5** shows that both depression and anxiety scores reduced significantly at the end of the intervention, among those who had a score ≥10 on the depression/anxiety scales at the beginning of the intervention, as identified by ASHAs. After adjusting for clustering, there was a significant reduction in score of depression by 3.6 (SE 0.6, *P* < 0.0001) and a reduction in anxiety score by 1.3 (SE 0.4, *P* = 0.004), at the end of intervention.

**Table 5:** Change in depression/anxiety scores for those who had a score ≥10 at the beginning of the intervention as screened by ASHAs

Depression score					
**Descriptive statistic response**	**Beginning of intervention**	**Post intervention**	Test statistics*		***P*–value**
**Depression score:**					
N	73	69			
Mean (SD)	13.84 (4.14)	4.59 (5.35)			
Median	12	3			
Minimum	10	0			
Maximum	27	26			
**Change in depression score:**					
n†		69	–11.92		<.0001
Mean (SD)		–9.20 (6.41)			
Median		–9			
Min		–24			
Max		8			
**Anxiety score:**					
N	31	30			
Mean (SD)	12.42 (2.84)	3.73 (3.61)			
Median	12	3			
Minimum	10	0			
Maximum	21	15			
**Change in anxiety score:**					
n†		30	–9.562	<.0001	
Mean (SD)		–8.77 (5.02)			
Median		–9			
Minimum		–21			
Maximum		3			

*Paired t–test.

†Number of individuals who were assessed at both points in time.

The results from the anti–stigma campaign show that during the project period, the communities’ knowledge, attitude and behavior related to mental health [26] had consistently shown an improved lower score, especially for the attitude and behavior related questions (**Table 6** and Table S1 in **Online Supplementary Document(Online Supplementary Document)**). The responses on the BACE–TS [27] are indicative of low baseline stigma which reduced even further during the project period (**Table 7** and Table S2 in **Online Supplementary Document(Online Supplementary Document)**).

**Table 6:** Change in mean scores for Knowledge, Attitude, and Behavior Questions from baseline to post–intervention*

Question	Mean (SD), baseline, n	Mean (SD), post intervention, n	Difference of mean (SD), n	*P*–value†
**Knowledge:**				
Mentally ill people tend to be violent	2.2 (1.34), 4401	1.6 (0.86), 193	–0.5 (1.57), 167	<.001
People with mental illness cannot live a good, rewarding life	1.9 (1.14), 4660	1.4 (0.68), 211	–0.5 (1.34), 192	<.001
People with severe mental health problems can fully recover	2.1 (1.25), 4694	1.7 (0.9), 215	–0.2 (1.43), 189	0.020
Medication can be an effective treatment for people with mental health problems.	1.6 (1.01), 4800	1.5 (0.68), 223	–0.2 (1.29), 204	0.067
**Attitude:**				
Mentally ill people shouldn’t get married	2.2 (1.4), 4591	1.4 (0.71), 205	–0.7 (1.62), 176	<.001
People with mental health problems are far less of a danger than most people suppose	1.8 (1.06), 4714	1.4 (0.65), 207	–0.5 (1.21), 191	<.001
We need to adopt a far more tolerant attitude toward people with mental illness in our society	1.6 (0.97), 4861	1.3 (0.64), 227	–0.2 (1.11), 215	0.013
People with mental health problems should not be given any responsibility	1.9 (1.22), 4798	1.5 (0.81), 222	–0.6 (1.58), 207	<.001
**Behavior:**				
If you suffered from a mental health problem would you tell your family or friends?	2.8 (0.61), 5167	2.8 (0.59), 232	0.1 (0.84), 232	0.139
I would be willing to live with someone with a mental health problem	2 (1.37), 4858	2.1 (1.3), 224	0.1 (1.89), 213	0.328
I would be willing to work with someone with a mental health problem	2 (1.26), 4862	1.6 (0.84), 224	–0.3 (1.51), 213	0.004
I would be willing to live nearby someone with a mental health problem	1.9 (1.22), 4862	1.6 (0.9), 223	–0.3 (1.57), 213	0.004
I would be willing to continue a relationship with a friend who developed a mental health problem	1.8 (1.11), 4857	1.5 (0.82), 227	–0.3 (1.42), 213	<.001

*Lower scores on the Knowledge, Attitude and Behavior questionnaire indicate that respondents are more agreeable to the statement.

†Difference in means includes only paired observation; *P*–value is calculated using paired t–test; n = observations for each analysis.

**Table 7:** Change in mean scores for each barrier in the Barriers to Access to Care Evaluation – Treatment Stigma Subscale, from baseline to post–intervention*

Question	Mean (SD) Baseline, n	Mean(SD) Post Intervention, n	Difference of Mean (SD), n	*P-value
Concern that I might be seen as weak for having a mental health problem	0.17 (0.45), 4416	0.07 (0.27), 232	0.16 (0.55), 216	<.0001
Concern that it might harm my chances when applying for jobs	0.09 (0.35), 1690	0.06 (0.24), 17	0 (0), 10	Not computed
Concern about what my family might think, say, do or feel	0.17 (0.45), 4416	0.13 (0.36), 232	0.1 (0.64), 216	0.03
Feeling embarrassed or ashamed	0.13 (0.38), 4416	0.09 (0.30), 232	0.09 (0.56), 216	0.02
Concern that I might be seen as crazy	0.13 (0.38), 4416	0.13 (0.37), 232	0.02 (0.59), 216	0.56
Concern that I might be seen as a bad parent	0.12 (0.38), 3848	0.1 (0.34), 218	0.08 (0.54), 193	0.04
Concern that people I know might find out	0.13 (0.40), 4416	0.1 (0.33), 232	0.07 (0.59), 216	0.08
Concern that people might not take me seriously if they found out I was having professional care	0.12 (0.39), 4416	0.13 (0.41), 232	0.02 (0.63), 216	0.66
Not wanting a mental health problem to be on my medical records	0.13 (0.43), 4416	0.3 (0.79), 232	-0.18 (0.84), 216	0.002
Concern that my children may be taken into care or that I may lose access or custody without my knowledge	0.12 (0.36), 3795	0.23 (0.71), 218	-0.05 (0.8), 194	0.37
Concern about what my friends might think, say or do	0.15 (0.40), 4416	0.18 (0.46), 232	-0.03 (0.61), 216	0.44
Concern about what people at work might think, say or do	0.14 (0.39), 4416	0.13 (0.38), 232	0.03 (0.55), 216	0.45
Overall mean	0.14 (0.27), 4416	0.14 (0.31), 232	0.03 (0.45), 216	0.39

*Lower scores on the BACE–TS suggest that the barrier is perceived less of an issue or none at all.

**†***P–*value is calculated using paired t–test; n = observations for each analysis.

## DISCUSSION

We found that about 5% individuals suffered from common mental health disorders in a rural, remote community in India. This population is considered to be particularly vulnerable due to remote location making traveling for help–seeking difficult, poorer health facilities and limited mental health facilities. The rates of CMD are somewhat lower than reported in a recent national mental health survey from India found that in community settings, CMD prevalence is around 10%, and it includes substance use disorders [29]. The SMART Mental Health Project did not include substance use disorders under its definition for CMD. Moreover it included only moderate to severe depression/anxiety, hence the rates were even lower. If we Include mild depression/anxiety, the prevalence for CMD increases to 15%. This is similar to the lower value observed in earlier studies, where the prevalence of CMD was estimated to be between 13–50% [2], with the variability explained due to different study designs and instruments.

We implemented a mobile technology enabled mental health services model coupled with training of primary care health workers with the aim of increasing identification and referral of community members with CMD. We found the intervention of training, task shifting, and referral model to be feasible, acceptable to community and health care providers. It also led to identification of people with CMD in this rural community. Detailed qualitative process evaluation will be reported subsequently and will provide more details about feasibility and acceptability, but the fact that these could be implemented in this population and the intervention could be delivered using a predetermined strategy, provides preliminary indication about the feasibility and acceptability of the intervention. It appeared to lead to an increase in the proportion of individuals seeking mental health services. Few studies from India and other LMICs have focused on the mental health issues of ST communities leading to limited knowledge about the prevalence of mental disorders or availability of mental health services in such communities. Remoteness of ST areas and difficulties in conducting research are some of the reasons for this. Studies show that ST populations seek care overwhelmingly through public sector, which highlights the importance of strengthening the public health sector in such areas [7]. To the best of our knowledge, this is the first project from India which reports on using innovative mobile–technology based mental health service delivery mechanisms in an ST area.

The project has a number of limitations. First, this is a pre–post design with no controls, hence the results need to be interpreted cautiously. Second, only 38 out of the screen positive individuals identified by interviewers at baseline were also identified by ASHAs. This suggests a poor inter–rater reliability. However, the reasons for such could be that some of the initial screen positive have had spontaneous remission. A systematic review showed that for major depressive disorder 23% have spontaneous remission within 3 months, and mild–to moderate depression has 20–30% higher remission than severe forms [30]. The time difference for the two assessments in this project was 2–3 months, and a number of screen positive individuals were suffering mild to moderate depression with suicidal risk. Another explanation is the ‘retest effect’ where results from psychiatric research show that retesting using the same instrument can lead to attenuated results due to a number of reasons [31]. Additionally, preliminary information from qualitative interviews of different stakeholders conducted at post–intervention stage reveal that some community members had not disclosed their symptoms to ASHAs due to apprehensions about the type of treatment they would be asked to undertake; fear that disclosing mental health symptoms to ASHAs could be inadvertently leaked within their community as ASHAs are part of the community; and lack of confidence in the PHC doctor’s ability to manage mental disorders (data not shown). Third, the 3–month intervention period was short and allowed only 30 screen positive individuals to access services. Our experience gained through ongoing research in other villages show that uptake of services increases gradually with time and 3 months was insufficient. The short time also prevented organizing more than 3 health camps, which were the main avenues to seek care by the community as they preferred to receive care closer to home which helped to reduce their traveling time and cost. Fourth, the short period of intervention also implies that while service contact information is available, information about adequacy and effectiveness of treatment is not available. While there was significant changes in depression/anxiety score, the sample size was small, making it difficult to generalize the results. This could also represent natural remission and regression to mean. Finally, though there was a need for anti–depressants in only one case, the lack of psychotropic medications at primary health center meant that the individual had to be referred to the district hospital.

More women were identified as screen positive compared to men and reflects the higher prevalence of depression in women found by others [32–34]. However, another reason could be that due to cultural norms, men are less likely to express symptoms of depression or anxiety leading to under–reporting [35]. Marital status, education, and age were significantly associated with CMD, as had been observed earlier [32]. Financial problems, illness/injury to self or to someone in the family, and death of a loved one were the commonest stressors associated with moderate/severe depression, similar to earlier research [32]. Lifetime alcohol use had a high prevalence and some reasons suggested for higher consumption among tribal populations are increased poverty, illiteracy, increased stress, and peer pressure [36].

The SMART Mental Health project used three key strategies to provide mental health care. The first involved task shifting, where ASHAs and PHC doctors were trained in screening and managing CMD. The second involved developing and implementing a mobile technology enabled EDSS for use by ASHAs and doctors, to screen, diagnose, and manage CMD. The third involved implementation of an anti–stigma campaign.

### Task–shifting

Task shifting combined with health system restructuring is effective for management of non–communicable disorders, though its cost–effectiveness is still inconclusive [8]. Task shifting is particularly relevant for mental health care in resource poor settings with few mental health professionals [37], and was reported to be effective and acceptable in an earlier study done in tribal areas [38]. But in that study majority of cases who sought treatment suffered from severe mental disorders, which are easier to identify in the community, hence more likely to be treated when compared to CMD [39]. Our project showed that ASHAs and PHC doctors were able to perform the task of screening and managing CMD using the EDSS.

### EDSS enabled mental health services delivery model

Using the EDSS the ASHAs were able to screen and refer individuals with CMD to the PHC doctors, who in turn used an EDSS with the mhGAP–IG tool to diagnose and manage such individuals. Overall there was an increase in accessing mental health care from PHC doctors. The results also appeared to be beneficial and showed a significant decrease in the depression/anxiety scores at post–intervention, for those individuals who had scored moderate–severe depression at the beginning of the intervention. The results showed that a mobile–technology based mental health services delivery strategy can be implemented in this community using existing government resources.

This project helps to provide mental health services in rural and remote areas and provides some preliminary evidence suggesting that task shifting coupled with a mobile technology enabled EDSS can be used is such areas after suitable adaptations. mHealth enabled health care models have been criticized [40] for a lack of appropriate scaled up projects to follow the initial pilot projects. The aim of this project was to use the learnings from a proof–of–concept project to develop more robust studies which could be scaled up across larger areas. The EDSS provided a platform that not only helped ASHAs to screen for CMD, but also allowed them to follow them up to ensure treatment adherence. It also enabled the doctors to use an evidence–based guideline to manage the cases. One review identified EDSS as an important strategy to improve health care delivery, especially where algorithms were used to provide treatment plans and included patient and provider prompts [14]. However, another review found that data from LMICs is limited, but a number of ongoing projects with a potential to provide valuable information about mHealth solutions in health care are available [41], and this project has the same potential.

### Anti–stigma campaign

Responses from the Knowledge, Attitude and Behavior questionnaire (**Table 6**) show that about 20% of the population knew someone with mental disorders and that may be a reason for the low level of baseline stigma in the population. About 7% of people responded that faith healers and religious leaders were the first point of contact for people with mental illness and this has been reported earlier from India [42]. During the intervention period people’s attitudes and behaviors appeared to improve but not their scores on knowledge, and this is similar to other studies [17]. Stigma related to help–seeking was low and is similar to earlier research from LMIC [43].

## CONCLUSIONS

In conclusion, the SMART Mental Health project showed that the delivery of mobile based mental health services was possible in the community and preliminary evidence suggests an increase in mental health service use. Future research needs to use more robust randomized controlled trial methods to identify the effectiveness and cost–effectiveness of the program. In order to take this to scale, there needs to be greater involvement of the government at all levels and systems should be in place at PHCs to enable mental health services delivery including training of staff, provision of psychotropic medications, basic counselling services and a streamlined referral systems to the next level where specialist mental health services can be provided to those in need.
