# Supplementary material for: Increasing use of mental health services in remote areas using mobile technology: a pre–post evaluation of the SMART Mental Health project in rural India
Source: J Glob Health. 2017 Mar 18;7(1):010408. doi: 10.7189/jogh.07.010408 (PMC5370210; doi:10.7189/jogh.07.010408)
Supplement: Online Supplementary Document [file jogh-07-010408-s001.pdf]

Online Supplementary Document

Maulik et al. Increasing use of mental health services in remote areas using mobile technology: a pre–post evaluation of the SMART Mental Health project in rural India

J Glob Health 2017;7:010408

Table S1: Summary of Knowledge, Attitude and Behaviour

	Question	Response	Baseline (N=5167) n(%)	Post Intervention (N=232) n(%)
Context*	Have you seen or heard any information about mental health or mental illness issues in the last year, in any of these ways? (Choose all that apply)	Newspaper TV People talking about it		8(3.45)
		TV	2255(43.64)	119(51.29)
		Other (specify)	681(13.18)	
		People talking about it	1243(24.06)	79(34.05)
		TV People talking about it	385(7.45)	13(5.60)
		Newspaper TV	144(2.79)	5(2.16)
	Where do people in this community first go to seek care for mental illness?	Hospital	3953(76.50)	153(65.95)
		Local clinic	757(14.65)	75(32.33)
		Traditional healer	201(3.89)	3(1.29)
		Religious or spiritual advisor	164(3.17)	1(0.43)
		Nowhere/care is not available	46(0.89)	1(0.43)
	Do any of the following people you know have a mental illness?	Friend/Acquaintance	80(1.55)	3(1.29)
		No one known	4005(77.51)	210(90.52)

	Question	Response	Baseline (N=5167) n(%)	Post Intervention (N=232) n(%)
		Neighbour	766(14.82)	13(5.60)
		Family member in this household	102(1.97)	2(0.86)
		Family member outside this household	162(3.14)	4(1.72)
Knowledge	Mentally ill people tend to be violent	Agree strongly	1677(32.46)	108(46.55)
		Agree slightly	1492(28.88)	77(33.19)
		Neither agree nor disagree	431(8.34)	
		Disagree slightly	254(4.92)	
		Disagree strongly	547(10.59)	8(3.45)
		Don’t know	766(14.82)	39(16.81)
	People with mental illness cannot live a good, rewarding life.	Agree strongly	2207(42.71)	135(58.19)
		Agree slightly	1498(28.99)	70(30.17)
		Neither agree nor disagree	416(8.05)	2(0.86)
		Disagree slightly	267(5.17)	1(0.43)
		Disagree strongly	272(5.26)	3(1.29)
		Don’t know	507(9.81)	21(9.05)
	People with severe mental health problems can fully recover.	Agree strongly	1984(38.40)	91(39.22)
		Agree slightly	1525(29.51)	111(47.84)
		Neither agree nor disagree	588(11.38)	2(0.86)
		Disagree slightly	109(2.11)	1(0.43)
		Disagree strongly	488(9.44)	10(4.31)
		Don’t know	473(9.15)	17(7.33)
	Medication can be an effective treatment for people with mental health problems.	Agree strongly	2874(55.62)	132(56.90)
		Agree slightly	1333(25.80)	77(33.19)
		Neither agree nor disagree	225(4.35)	12(5.17)
		Disagree slightly	171(3.31)	
		Disagree strongly	197(3.81)	2(0.86)

	Question	Response	Baseline (N=5167) n(%)	Post Intervention (N=232) n(%)
		Don't know	367(7.10)	9(3.88)
Attitude	Mentally ill people shouldn't get married	Agree strongly	2112(40.87)	139(59.91)
		Agree slightly	1100(21.29)	58(25.00)
		Neither agree nor disagree	475(9.19)	3(1.29)
		Disagree slightly	322(6.23)	2(0.86)
		Disagree strongly	582(11.26)	3(1.29)
		Don't know	576(11.15)	27(11.64)
	People with mental health problems are far less of a danger than most people suppose.	Agree strongly	2438(47.18)	138(59.48)
		Agree slightly	1449(28.04)	60(25.86)
		Neither agree nor disagree	416(8.05)	7(3.02)
		Disagree slightly	216(4.18)	
		Disagree strongly	195(3.77)	2(0.86)
		Don't know	453(8.77)	25(10.78)
	We need to adopt a far more tolerant attitude toward people with mental illness in our society.	Agree strongly	3234(62.59)	168(72.41)
		Agree slightly	1077(20.84)	44(18.97)
		Neither agree nor disagree	177(3.43)	12(5.17)
		Disagree slightly	247(4.78)	3(1.29)
		Disagree strongly	126(2.44)	
		Don't know	306(5.92)	5(2.16)
	People with mental health problems should not be given any responsibility.	Agree strongly	2422(46.87)	135(58.19)
		Agree slightly	1249(24.17)	70(30.17)
		Neither agree nor disagree	419(8.11)	11(4.74)
		Disagree slightly	400(7.74)	1(0.43)
		Disagree strongly	308(5.96)	5(2.16)
		Don't know	369(7.14)	10(4.31)

	Question	Response	Baseline (N=5167) n(%)	Post Intervention (N=232) n(%)
Behaviour	If you suffered from a mental health problem would you tell your family or friends?	No one	495(9.58)	21(9.05)
		Friends	265(5.13)	5(2.16)
		Family	4407(85.29)	206(88.79)
	I would be willing to live with someone with a mental health problem.	Agree strongly	2351(45.50)	90(38.79)
		Agree slightly	1412(27.33)	89(38.36)
		Neither agree nor disagree	260(5.03)	15(6.47)
		Disagree slightly	206(3.99)	
		Disagree strongly	629(12.17)	30(12.93)
		Don’t know	309(5.98)	8(3.45)
	I would be willing to work with someone with a mental health problem	Agree strongly	2345(45.38)	115(49.57)
		Agree slightly	1573(30.44)	86(37.07)
		Neither agree nor disagree	212(4.10)	17(7.33)
		Disagree slightly	288(5.57)	
		Disagree strongly	444(8.59)	6(2.59)
		Don’t know	305(5.90)	8(3.45)
	I would be willing to live nearby someone with a mental health problem	Agree strongly	2401(46.47)	126(54.31)
		Agree slightly	1544(29.88)	73(31.47)
		Neither agree nor disagree	240(4.64)	15(6.47)
		Disagree slightly	297(5.75)	2(0.86)
		Disagree strongly	380(7.35)	7(3.02)
		Don’t know	305(5.90)	9(3.88)
	I would be willing to continue a relationship with a friend who developed a mental health problem.	Agree strongly	2674(51.75)	151(65.09)
		Agree slightly	1440(27.87)	60(25.86)
		Neither agree nor disagree	228(4.41)	8(3.45)
		Disagree slightly	254(4.92)	3(1.29)
		Disagree strongly	261(5.05)	5(2.16)
		Don’t know	310(6.00)	5(2.16)

	Question	Response	Baseline (N=5167) n(%)	Post Intervention (N=232) n(%)

*A subject can be counted more than once for context questions and only top 5 responses are reported

Table S2: Change in mean scores for each barrier in the Barriers to Access to Care Evaluation - Treatment Stigma Subscale

Question	Response	Baseline (N=4416) n(%)	Post intervention (N=232) n(%)
Concern that I might be seen as weak for having a mental health problem	Not at all	3804(86.14)	217(93.53)
	A little	483(10.94)	14(6.03)
	Quite a lot	125(2.83)	1(0.43)
	A lot	4(0.09)	0(0.00)
Concern that it might harm my chances when applying for jobs	Not at all	1566(35.46)	16(6.90)
	A little	94(2.13)	1(0.43)
	Quite a lot	29(0.66)	0(0.00)
	A lot	1(0.02)	0(0.00)
	NA	2726(61.73)	215(92.67)
Concern about what my family might think, say, do or feel	Not at all	3786(85.73)	205(88.36)
	A little	513(11.62)	25(10.78)
	Quite a lot	111(2.51)	2(0.86)
	A lot	6(0.14)	0(0.00)
Feeling embarrassed or ashamed	Not at all	3916(88.68)	213(91.81)
	A little	434(9.83)	18(7.76)
	Quite a lot	61(1.38)	1(0.43)
	A lot	5(0.11)	0(0.00)
Concern that I might be seen as crazy	Not at all	3939(89.20)	206(88.79)
	A little	400(9.06)	23(9.91)
	Quite a lot	75(1.70)	3(1.29)
	A lot	2(0.05)	0(0.00)
Concern that I might be seen as a bad parent	Not at all	3429(77.65)	200(86.21)
	A little	361(8.17)	15(6.47)
	Quite a lot	55(1.25)	3(1.29)
	A lot	3(0.07)	0(0.00)
	NA	568(12.86)	14(6.03)

Question	Response	Baseline (N=4416) n(%)	Post intervention (N=232) n(%)
Concern that people I know might find out	Not at all	3940(89.22)	210(90.52)
	A little	382(8.65)	20(8.62)
	Quite a lot	90(2.04)	2(0.86)
	A lot	4(0.09)	0(0.00)
Concern that people might not take me seriously if they found out I was having professional care	Not at all	4010(90.81)	209(90.09)
	A little	308(6.97)	18(7.76)
	Quite a lot	94(2.13)	4(1.72)
	A lot	4(0.09)	1(0.43)
Not wanting a mental health problem to be on my medical records	Not at all	3979(90.10)	198(85.34)
	A little	304(6.88)	11(4.74)
	Quite a lot	125(2.83)	11(4.74)
	A lot	8(0.18)	12(5.17)
Concern that my children may be taken into care or that I may lose access or custody without my	Not at all	3376(76.45)	195(84.05)
	A little	380(8.61)	5(2.16)
	Quite a lot	38(0.86)	9(3.88)
	A lot	1(0.02)	9(3.88)
	NA	621(14.06)	14(6.03)
Concern about what my friends might think, say or do	Not at all	3851(87.21)	196(84.48)
	A little	490(11.10)	31(13.36)
	Quite a lot	74(1.68)	4(1.72)
	A lot	1(0.02)	1(0.43)
Concern about what people at work might think, say or do	Not at all	3889(88.07)	204(87.93)
	A little	450(10.19)	25(10.78)
	Quite a lot	77(1.74)	3(1.29)
